# Ultrahigh-Density Linkage Map Construction Using Low-Coverage Whole-Genome Sequencing of a Doubled Haploid Population: Case Study of Torafugu (*Takifugu rubripes*)

**DOI:** 10.3390/genes9030120

**Published:** 2018-02-26

**Authors:** Xiang Zhang, Misaki Mizukoshi, Hong Zhang, Engkong Tan, Yoji Igarashi, Yutaka Suzuki, Susumu Mitsuyama, Shigeharu Kinoshita, Kazuyoshi Saito, Shugo Watabe, Shuichi Asakawa

**Affiliations:** 1 Department of Aquatic Bioscience, Graduate School of Agricultural and Life Sciences, The University of Tokyo, Bunkyo, Tokyo 113-8657, Japan; zhangxiangjp@gmail.com (X.Z.); waterover6101ocean@gmail.com (M.M.); zhanghon@umich.edu (H.Z.); tanengkong@gmail.com (E.T.); aiga@mail.ecc.u-tokyo.ac.jp (Y.I.); mituyama@dmb.med.keio.ac.jp (S.M.); akino@mail.ecc.u-tokyo.ac.jp (S.K.); 2 Department of Medical Genome Sciences, Graduate School of Frontier Sciences, The University of Tokyo, Kashiwa, Chiba 277-8562, Japan; ysuzuki@k.u-tokyo.ac.jp; 3 Akita Prefectural Fisheries Promotion Center, Oga, Akita 010-0531, Japan; kazu-s@pref.akita.lg.jp; 4 School of Marine Bioscience, Kitasato University, Sagamihara, Kanagawa 252-0373, Japan; swatabe@kitasato-u.ac.jp

**Keywords:** genetic linkage map, low-coverage whole-genome sequencing, doubled haploid population, *Takifugu rubripes*, linkage phase unknown

## Abstract

Next-generation sequencing enables genome-wide genotyping of a large population and further facilitates the construction of a genetic linkage map. Low-coverage whole-genome sequencing has been employed for genetic linkage map construction in several species. However, this strategy generally requires available high-quality reference genomes and/or designed inbred pedigree lines, which restrict the scope of application for non-model and unsequenced species. Here, using torafugu (*Takifugu rubripes*) as a test model, we propose a new strategy for ultrahigh-density genetic linkage map construction using low-coverage whole-genome sequencing of a haploid/doubled haploid (H/DH) population without above requirements. Low-coverage (≈1×) whole-genome sequencing data of 165 DH individuals were used for de novo assembly and further performed single nucleotide polymorphisms (SNPs) calling, resulting in the identification of 1,070,601 SNPs. Based on SNP genotypes and de novo assembly, genotypes were associated with short DNA segments and an ultrahigh-density linkage map was constructed containing information of 802,277 SNPs in 3090 unique positions. Comparative analyses showed near-perfect concordance between the present linkage map and the latest published torafugu genome (FUGU5). This strategy would facilitate ultrahigh-density linkage map construction in various sexually reproducing organisms for which H/DH populations can be generated.

## 1. Introduction

A genetic linkage map is a powerful tool in genetic and genomic research. It lays a strong foundation for comparative genomics and provides vital clues toward understanding genome evolution and divergence [1,2,3,4]. Moreover, it facilitates genotype–phenotype association mapping and enables investigating the genetics of complex phenotypic traits [5,6,7]. It also contributes toward characterization of genome structure and serves as the backbone for anchoring unplaced/misplaced scaffolds for chromosome-scale assembly [8,9,10,11].

Owing to the rapid development of next-generation sequencing (NGS) in the last decade, the ability to simultaneously sequence a large number of individuals in a multiplex manner has now become possible, so that an entire population can be rapidly genotyped for linkage mapping [12]. Since 2010, low-coverage whole-genome resequencing has been employed for construction of the genetic linkage maps of rice [13], shiitake mushroom [14], and safflower [15]. However, all of these cases relied on the availability of high-quality reference genome sequences and/or designed inbred pedigree lines to carry out the linkage mapping prior to resequencing of the mapping population. This requirement currently limits the wide application of low-coverage whole-genome resequencing in non-model organisms, especially for those with unexplored genomes.

To resolve this issue, we hypothesized that an ultrahigh-density genetic linkage map could be constructed using low-coverage whole-genome sequencing of haploid/doubled-haploid (H/DH) population without requiring a high-quality reference genome and the laborious establishment of inbred lines. H/DH individuals can be generated via natural or artificial uniparental reproduction, which is found in a wide range of species in several kingdoms. Indeed, in recent years, the H/DH population has been exploited as an ideal population type for genetic linkage map construction, particularly in plants [16,17,18,19,20] and teleosts [21,22,23,24] due to their well-developed H/DH technologies [25,26]. The main advantage of H/DH individuals for genotype sequencing is that a relatively low sequencing coverage is sufficient without loss of accuracy compared to the coverage necessary for sequencing more common diploid individuals owing to the presence of heterozygous single nucleotide polymorphisms (SNPs). To test this strategy, we established a DH population of the wild pufferfish or torafugu (*Takifugu rubripes*) through gynogenesis.

Torafugu is a popular species with economic importance in the waters of East Asia, and has emerged as an ideal model in genomic studies owing to its compact genome [27]. In fact, the torafugu genome is considered to be one of the smallest (≈400 Mb) among vertebrates and is approximately eight times smaller than the human genome [27]. Another advantage of torafugu as a model for genetic analysis is its similarity to mammals, including a shared body plan and physiological systems. Thus, the compact genome can favor the discovery of genes and gene regulatory regions with clear counterparts in the human genome, and the torafugu genome can further serve as a reference to understand the structure, function, and evolution of vertebrate genomes [28,29]. In the most recent fifth version of the torafugu genome assembly (FUGU5), 72% of the scaffolds have been located and oriented after integration with the torafugu genetic linkage map, comprising 1220 microsatellite markers; however, the remaining 14% have been located but not oriented, and the other 14% have not yet been assigned [30]. Therefore, the construction of a higher-density genetic linkage map of torafugu is needed to be able to expand the contiguity and improve the quality of the genome assembly. 

Based on the proposed strategy, an ultrahigh-density linkage map of torafugu was constructed. The accuracy of the obtained linkage map was validated with comparison to the published genome FUGU5. The proposed strategy represents a cost-effective and less complex tool for genetic linkage map construction and can be widely applied in a wide diversity of sexual organisms, especially non-model and unsequenced species, for which H/DH populations can be generated.

## 2. Materials and Methods

### 2.1. Preparation of a Doubled-Haploid Population of Torafugu

A wild female torafugu was purchased from a market in Akita Prefecture and was subjected to mito-gynogenesis for generating a DH population according to the process described in detail in our previous paper [31]. In brief, mature oocytes were fertilized with inactive sperm of a male torafugu (from the same market) that had been pretreated with ultraviolet radiation at 40 mJ/cm^2^. After fertilization for 3 h, the eggs were subjected to 45 min of cold-shock treatment at 0.6 °C, followed by incubation in aerated tanks with fresh seawater at 18.0 °C. Several days after artificial insemination, hundreds of eggs were observed to contain embryonic bodies, which were selected for further analysis.

### 2.2. Whole-Genome Sequencing 

Genomic DNA was extracted from each of the selected 192 eggs using Agencourt DNAdvance Kit (Beckman Coulter Genomics, Beverly, MA, USA) after homogenization. An average of 125 ng DNA was obtained from each sample. DNA libraries of these individuals were prepared and barcoded according to the Nextera DNA Library Prep Reference Guide (Illumina, San Diego, CA, USA) and were then subjected to sequencing in two lanes of Illumina HiSeq 2000 system. A total of 74.43 Gb of sequencing data, consisting of 2 × 100-bp paired-end reads with an average insert size of 230 bp, were obtained from 192 samples of the generated DH torafugu population. Potential remnants of adapter sequences were removed, low-quality bases with a Phred quality score below 20 were trimmed, and the 23 samples with very low sequencing coverage were removed. After these processes, a total of 71.32 Gb of sequencing data from 169 samples were ultimately reserved and applied to further analysis.

### 2.3. De Novo Assembly and SNP Calling

The obtained sequencing data were utilized to perform de novo assembly on the SOAPdenovo2 [32] assembler under a k-mer value of 58. The sequencing data of each sample were mapped to the obtained de novo assembly using Burrows–Wheeler Aligner (BWA) [33], followed by SNP calling using SAMtools with default parameters [34]. Four samples were identified as partial diploids due to the existence of many heterologous SNPs. Considering that DNA polymorphisms would have effects on de novo assembly, the sequencing data (1.74 Gb) from these four samples were removed. Thus, a total of 69.58 Gb sequencing data from 165 samples were reserved. According to the torafugu genome size (approximately 400 Mb), the total sequencing data coverage was estimated at 174, whereas the average coverage for each sample was 1.05 ± 0.76. The remaining sequencing data (69.58 Gb) were utilized to perform a second round of de novo assembly, and SNP calling was then performed for each sample with the same parameters.

### 2.4. Marker Genotype Scoring

**Figure 1:**
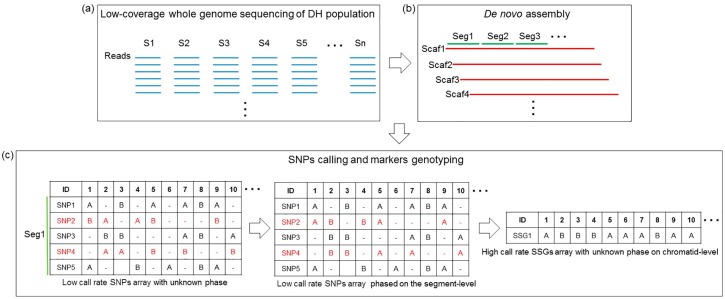
Flowchart of high-call-rate markers genotyping. (**a**) Low-coverage whole-genome sequencing of the doubled-haploid (DH) mapping population was performed to generate a library of short gun reads (in blue) for each sample (S1‒Sn). (**b**) De novo assembly was performed using the total whole-genome sequencing data to generate scaffolds (in red) containing a series of segments (in green). (**c**) Single nucleotide polymorphisms (SNP) calling and genotyping of each sample was carried out to construct a low-call-rate SNPs array (SNPs × Samples) with unknown phase. The SNPs (SNP1‒5) located on one short segment (Seg1) were phased at the segment level and merged together to assign the short segment genotype (SSG1). The high-call-rate SSGs array (SSGs × Samples) was then constructed with unknown phase at the chromatid level.

**Figure 2:**
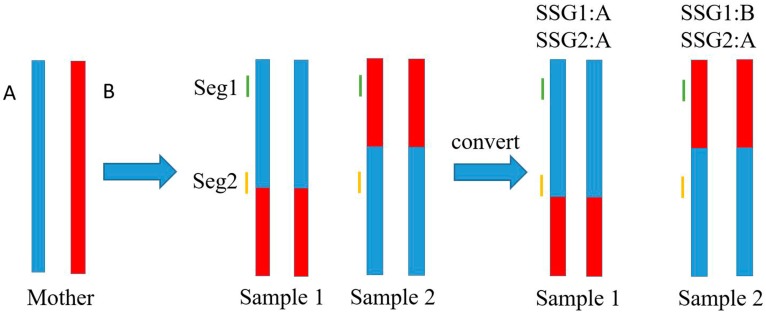
Schematic illustration of the conversion of SSGs under different circumstances. The maternal chromatids possess linkage phase score A (blue) and B (red). In Sample 1, recombination occurs on Seg2 but outside of Seg1. In Sample 2, recombination occurs outside of both Seg1 and Seg2, which is the most common case.

Owing to the low-coverage (≈1×) sequencing, most of the SNPs in each sample were detected once or less, which led to a large quantity of missed genotypes and insufficient data for genotyping calibration. As shown in Figure 1, we arbitrarily assigned a phase to every allele of each SNP (with one type scored as “A”, the other scored as “B”, and unknown scored as “-”) because of the unknown linkage phase. Subsequently, a low-call-rate SNP dataset of unknown phase was obtained. However, based on the contig/scaffold of the above de novo assembly, the genotype of adjacent SNPs could be testified and/or compensated by each other. The SNPs located on short DNA segments were phased at the segment level and merged together as a new genetic marker termed the short segment genotype (SSG). As shown in Figure 2, when recombination occurred outside of a short DNA segment (Seg1 in Sample 1, Seg1 and Seg2 in Sample 2), in most cases, the converted SSG possessed the same genotypes at the SNP sites within it. By contrast, when recombination occurred on a DNA segment such as Seg2 in Sample 1, the converted SSG was regarded to possess the same genotype as the genotype of SNPs dominating within the segment. In that case, the site of crossover was temporarily located to the terminal end of the DNA segment. Practically, this temporal operation would hardly influence the formation of the linkage map because the length of the DNA segment is much shorter than that of the entire chromosome. In addition, two crossover events hardly ever occur within such a short DNA segment. The maximum segment length of the SSGs was set to 8 kb, so that the SSGs (>0.9 call rate) could harbor as much genome-wide SNPs information as possible while maintaining the length of segments as short as possible (see details in Figure S1). A high-call-rate SSG dataset was generated and subjected to 0.9-call rate filtering. Each SSG was selected when it met the segregation ratio of around 1:1 in the population.

### 2.5. Construction of the Genetic Linkage Map

We used a modified version of an approach designed for phase-unknown genetic linkage mapping in ants to construct a genetic linkage map using the phase-unknown SSGs, described in brief as follows [35]. In the first step, the complete SSGs array was doubled for each dataset, and the genotype score of every doubled SSG was switched (i.e., convert “A” to “B” and “B” to “A”) so that a new dataset containing every possible linkage phase for each SSG in each individual was generated. In the second step, linkage groups were generated using the linkage analysis program MSTmap [36]. Since the new dataset was doubled, each linkage group was represented twice. In step three, one of the two identical linkage groups was discarded. Step four involved the detection of genotyping errors and conversion to unknown phase, considering that double recombination is a very unlikely event among close markers. In the fifth and final step, the accuracy of the order of SSGs in each linkage group was checked with the R package ‘ASMap’ [37].

### 2.6. Comparative Analyses between the Linkage Map and FUGU5

The sequences of the SSGs located on the genetic linkage map obtained with the strategy outlined above were aligned to the published torafugu genome (FUGU5) using BLAST (version 2.2.29) with an e-value cut-off of 1 × 10^−100^ and identity of at least 95%. Genomic synteny was visualized using CIRCOS 0.69 software [38]. The flanking sequences of all mapped SNPs (35 bp on either side) were also subjected to a BLAST search against the published genome with an e-value cut-off of 1 × 10^−26^.

## 3. Results

### 3.1. De Novo Assembly

In total, 69.58 Gb of sequencing data from 165 samples of the generated DH torafugu population were used to perform de novo assembly. The total sequencing data coverage was 174, whereas the average coverage for each sample was approximately 1. After performing de novo assembly using the sequencing data, a relative low-quality assembly of a total size of 356.59 Mb and N50 size of 22,235 bp was generated, which was composed of 54,127 scaffolds with the length ranging from 200 to 264,568 bp. 

### 3.2. Genetic Markers Genotyping

After SNP calling from the sequencing data of each DH individual, a total of 1,070,601 SNPs were discovered in the population using the above de novo assembly as reference. Despite the existence of a large quantity of missed genotypes and insufficient data for genotyping calibration due to the low-coverage (≈1×) sequencing of each sample, the genotypes of adjacent SNPs could be testified/compensated by each other based on the above de novo assembly. Therefore, as shown in Figure 1, the DNA sequences of the assembly were sliced into short segments. The information of SNPs located on each segment was combined and a genotype was assigned to each segment. This low-call-rate SNP dataset was then converted into a high-call-rate SSG dataset. After 0.9-call rate filtering, 37,398 SSGs containing information of 833,594 SNPs were retained and could be used for construction of the genetic linkage map (see details in Supplementary Materials). 

### 3.3. Ultrahigh-Density Genetic Linkage Map Construction for Torafugu

**Figure 3:**
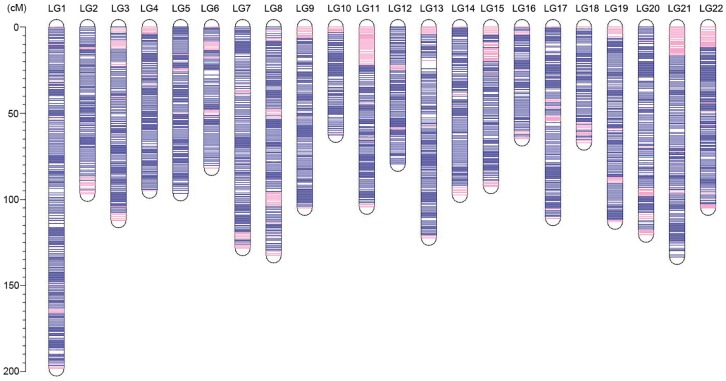
Ideograms of the genetic linkage map of torafugu. The genetic position of each SSG is illustrated in each linkage group. SSGs are indicated as a blue line if the sequences could be aligned to the published genome FUGU5 using BLASTN (version 2.2.29), and are otherwise shown as a pink line. LG: linkage group; the y-axis represents the genetic position (cM).

**Figure 4:**
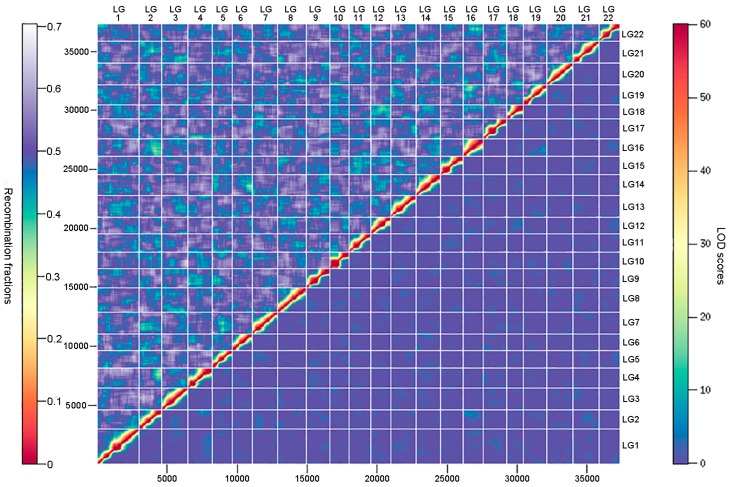
Heat map of recombination fractions (upper triangle) and logarithm of odds (LOD) scores (lower triangle) for all pairs of markers (SSGs) in the 22 linkage groups. LG represents linkage group.

An ultrahigh-density genetic linkage map (Figure 3) was constructed using the high-call-rate SSG dataset. As summarized in Table 1, the map consists of 37,343 SSGs in 3090 unique positions, containing the information of 802,277 SNPs (74.9% of total SNPs). The genetic linkage map contained 22 linkage groups, consistent with the number of chromosomes of the torafugu haploid genome. The genetic distances ranged from 62.75 cM of linkage group 10 (LG10) to 198.25 cM of LG1, with a total length of 2319.65 cM. Based on the unique marker positions, the estimated marker intervals ranged from 0.70 cM/marker in LG22 to 0.79 cM/marker in LG1, with an average marker interval of 0.75 cM/marker on the genetic linkage map. As shown in the heat map in Figure 4, the recombination fractions were considerably low between adjacent markers of each linkage group, indicating a low recombination frequency between them, whereas the LOD (logarithm of odds) scores between adjacent markers of each linkage group were high, indicating strong linkage between them. The 22 linkage groups appeared to be distinctly clustered.

### 3.4. Comparative Analyses

**Figure 5:**
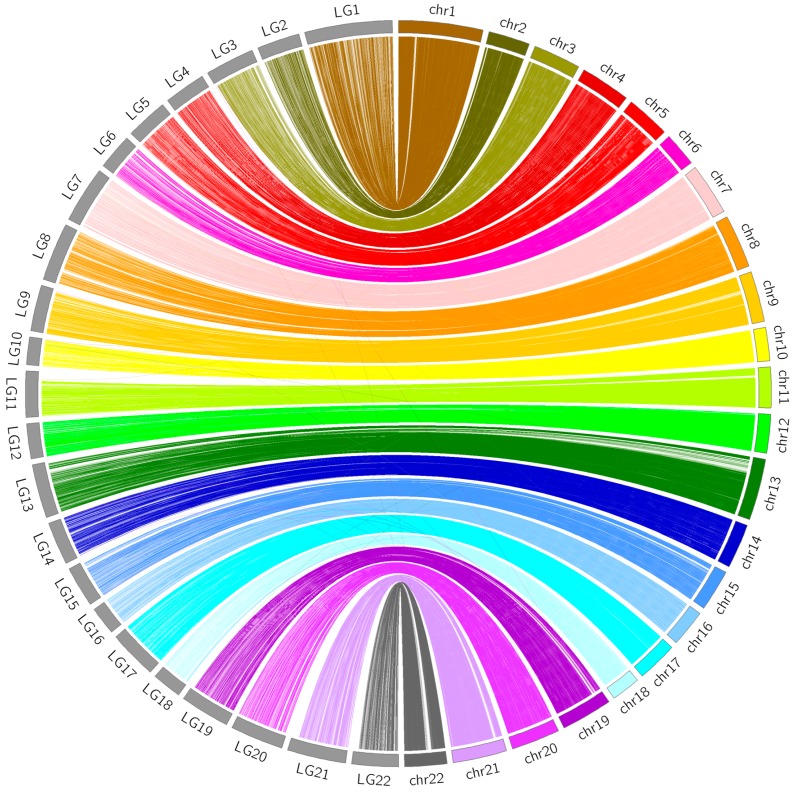
Comparison between the present genetic linkage map and FUGU5. Links connect the genetic and physical locations of each mapped SSG. Grey blocks on the left represent the 22 linkage groups of the present linkage map, whereas colorful blocks on the right represent the 22 chromosomes of FUGU5. LG: linkage group; chr: chromosome.

**Figure 6:**
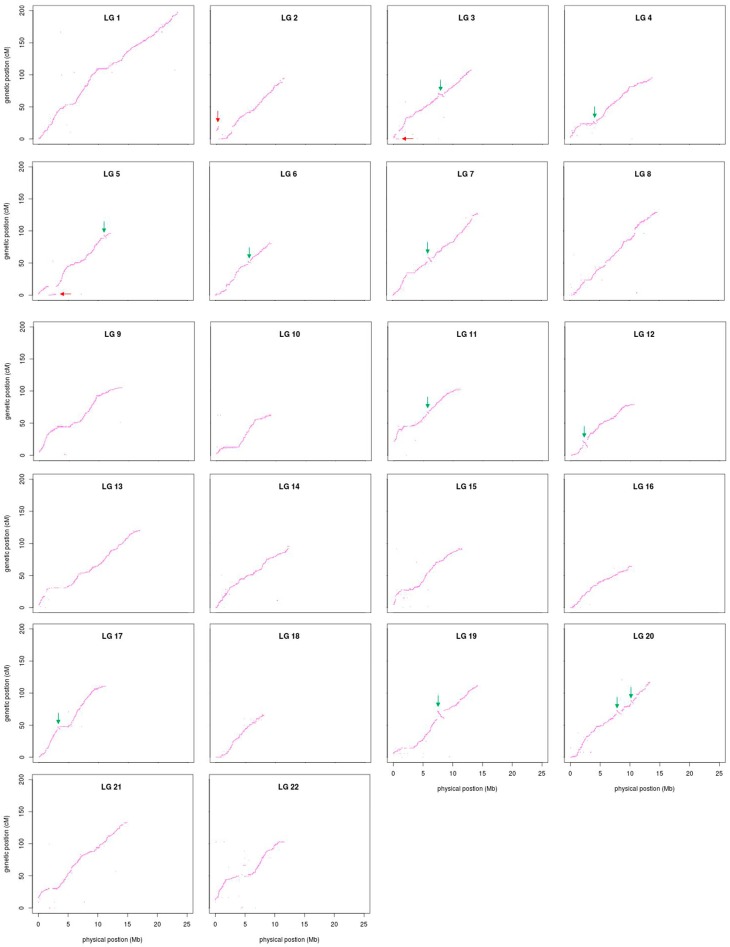
Concordance of SNP positions on the present genetic linkage map with those on FUGU5. The x-axis represents the physical position (Mb) of SNPs on FUGU5, whereas the y-axis represents the genetic position (cM) of SNPs on the linkage map. Red arrows indicate potentially misplaced or segmentally polymorphic regions, whereas green arrows indicate potentially mis-oriented or segmentally polymorphic regions in FUGU5.

The sequence information of the 37,343 SSGs of the linkage map obtained with the proposed strategy was subjected to BLASTN analyses against the latest published genome FUGU5. Overall, 31,822 SSGs could be mapped to the 22 chromosomes of FUGU5. As shown in Figure 5, the Circos plot indicated near-perfect concordance between the genetic and physical position of each matched SSG, and 5521 SSGs could be mapped to the 1583 (65.97 Mb) unassembled scaffolds. The genetic positions of these SSGs are also highlighted in Figure 3, indicating the regions where FUGU5 can be improved. Furthermore, 180 of these scaffolds (28.2 Mb) contained more than one unique genetic position, suggesting that they might be located on the chromosomes with direction. The flanking sequences of the SNPs contained in this genetic linkage map were also well aligned to FUGU5, and 532,424 SNPs mapped to the 22 chromosomes of the genome. The plot shown in Figure 6 reflects more detailed collinearity between the orders of the SNPs of each linkage group and each chromosome. The results also indicated possible mis-assembled regions or large segmental polymorphisms in chromosome 2, 3, 4, 5, 6, 7, 11, 12, 17, 19, and 20 of FUGU5.

## 4. Discussion

We successfully developed an effective strategy for the construction of an ultrahigh-density genetic linkage map of torafugu based on low-coverage (≈1×) whole-genome sequencing of each individual of a DH population generated through mito-gynogenesis. The sequencing data were used for de novo assembly and further SNP calling to generate a low-call-rate SNP dataset with unknown phase. Based on the relatively low-quality de novo assembly, an SSG was designed as a high-call-rate genetic marker to assign a genotype to a short DNA segment after combing the information of its constituent low-call-rate SNPs. The high-call-rate SSG dataset enabled the construction of an ultrahigh-density genetic linkage map containing most of the information of SNPs (sub-million in this case) of the mapping population. The accuracy of the present linkage map was verified by subsequent analyses of recombination fractions and assessment of LOD scores for all marker pairs, along with comparative analyses between the linkage map and FUGU5. In addition, integration with the present linkage map allowed for validation and further refinement of FUGU5. Based on these indicators, an improved genome assembly of torafugu will be achieved in our future work.

In the whole-genome assembly project, de novo assembly and linkage map construction are independent works contributing to chromosome-scale assembly. However, in our case, both of them can be achieved from the low-coverage whole-genome sequencing of the mapping population. Thus, for a non-sequenced species, de novo assembly, linkage map construction, and further chromosome-scale assembly can be efficiently completed by adopting our strategy.

The strategy of the present study was developed for genetic linkage map construction based on an H/DH dataset with phase-unknown format. This strategy would be ideally implemented for various types of sexually reproducing organisms that could be used to generate large numbers of H/DH individuals, especially plants, teleosts, and fungi, without requiring other complex crossing schemes and designed inbred pedigree lines to identify the linkage phase. Notably, our strategy based on SSG could be applied to the construction of an ultrahigh-density genetic linkage map using only single gamete cells, the ubiquitously existing haploids, combined with single-cell sequencing technology, which further extends the application range. One of the most important advantages of our strategy is that it does not require the parental genetic phases and/or a high-quality reference genome, which are necessary for existing single-gamete sequencing strategies [39,40,41,42] to achieve high-quality genetic mapping. 

The lack of a requirement of a high-quality reference genome for low-coverage whole-genome sequencing expands the application of our proposed strategy to a wide range of non-model and non-sequenced species. Moreover, our approach has an advantage of simplicity, in that whole-genome sequencing was applied to each individual sample, whereas existing techniques such as specific-locus amplified fragment sequencing (SLAF-seq) [43], reduced-representation libraries (RRLs) [44], and restriction-site-associated DNA sequencing (RAD-seq) [45] demand complicated processes for sequencing a small target portion of the whole genome. We successfully captured most of the SNPs of the population, which would allow for thoroughly characterizing complex genomes, whereas SLAF-seq, RAD-seq, and RRLs are only able to call a small portion of SNPs of the population. The low-coverage sequencing also makes the present strategy cost-effective. Collectively, these advantages demonstrate the potential of the present strategy as a good candidate for facilitating ultrahigh-density linkage map construction of various sexually reproducing organisms that can generate an H/DH population, and even can produce gametes combined with the single-cell sequencing technology platform.

## Figures and Tables

**Table 1:** Summary of the genetic linkage map of torafugu.

Linkage Group	Genetic Distance (cM)	SSGs Numbers	SNPs Numbers	Unique Positions	Marker Interval
LG1	198.25	2997	64,119	252	0.79
LG2	96.95	1588	34,369	128	0.76
LG3	112.13	1876	39,280	147	0.76
LG4	95.15	1722	35,535	132	0.72
LG5	96.42	1454	31,043	136	0.71
LG6	81.72	1431	31,616	110	0.74
LG7	128.42	1819	37,225	166	0.77
LG8	132.44	2055	44,302	174	0.76
LG9	104.88	1670	34,975	143	0.73
LG10	62.75	1396	30,320	82	0.77
LG11	104.43	1543	33,186	143	0.73
LG12	79.40	1408	30,636	110	0.72
LG13	122.28	1797	38,739	157	0.78
LG14	97.47	1770	37,750	128	0.76
LG15	92.43	1605	35,558	119	0.78
LG16	64.71	1417	30,875	89	0.73
LG17	110.91	1727	38,620	150	0.74
LG18	67.20	1157	26,774	89	0.76
LG19	112.95	1714	35,312	154	0.73
LG20	120.46	1830	38,989	152	0.79
LG21	133.31	1893	40,975	180	0.74
LG22	104.98	1474	32,079	149	0.70
Total	2319.65	37,343	802,277	3090	0.75 ^1^

^1^ Mean value of marker intervals.

## Supplementary Materials

The following are available online at http://www.mdpi.com/2073-4425/9/3/120/s1. Figure S1: Detailed information of the conversion of SNPs dataset to SSG dataset.
