# Supplementary material for: Ultrahigh-Density Linkage Map Construction Using Low-Coverage Whole-Genome Sequencing of a Doubled Haploid Population: Case Study of Torafugu (Takifugu rubripes)
Source: Genes (Basel). 2018 Feb 26;9(3):120. doi: 10.3390/genes9030120 (PMC5867841; doi:10.3390/genes9030120)
Supplement: Supplementary file 1 [file genes-09-00120-s001.pdf]

genes

MDPI

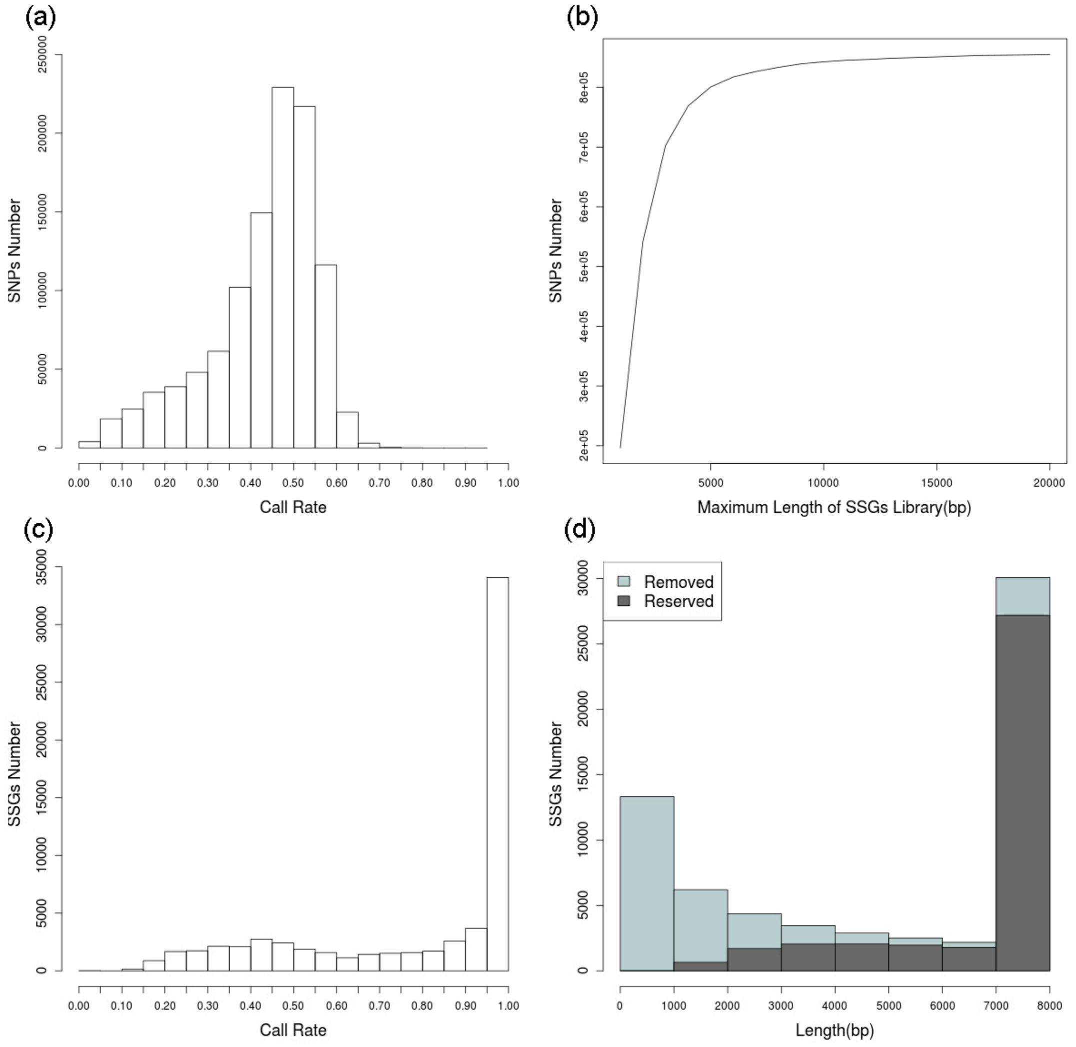

Figure S1. Detailed information of the conversion of SNPs dataset to SSGs dataset. (**a**) The distribution of SNPs under different call rates. The call rate of SNPs barely exceeded 0.65. (**b**) The number of SNPs contained in the SSGs library (>0.9 call rate) when the de novo assembly was sliced by 1000–20,000 bp. After 0.9-call rate filtering, the 20-kb SSGs library contained a total of 855,091 SNPs, whereas the 6-kb SSGs library contained 800,787 SNPs (>90% of the total) and the 8-kb SSGs library contained 826,595 SNPs (>95% of the total). (**c**) Distribution of SSGs under different call rates in the 8-kb SSGs library; the call rate of most of the SSGs exceeded 0.90. (**d**) Distribution of retained and removed SSGs under different lengths in the 8-kb SSGs library after 0.9-call rate filtering. Most of the shorter (1 kb) SSGs were removed, whereas most of the longer (8 kb) SSGs were retained.
